# Quantifying Reporting Timeliness to Improve Outbreak Control

**DOI:** 10.3201/eid2102.130504

**Published:** 2015-02

**Authors:** Axel Bonačić Marinović, Corien Swaan, Jim van Steenbergen, Mirjam Kretzschmar

**Affiliations:** National Institute for Public Health and the Environment (RIVM), Bilthoven, the Netherlands (A. Bonačić Marinović, C. Swaan, J. van Steenbergen, M. Kretzschmar);; University Medical Centre Utrecht, Utrecht, the Netherlands (A. Bonačić Marinović, M. Kretzschmar);; Leiden University Medical Centre, Leiden, the Netherlands (J. van Steenbergen)

**Keywords:** infectious disease, detection, notification, reporting, response, intervention, timeliness, bioterrorism and preparedness

## Abstract

The extent to which reporting delays should be reduced to gain substantial improvement in outbreak control is unclear. We developed a model to quantitatively assess reporting timeliness. Using reporting speed data for 6 infectious diseases in the notification system in the Netherlands, we calculated the proportion of infections produced by index and secondary cases until the index case is reported. We assumed interventions that immediately stop transmission. Reporting delays render useful only those interventions that stop transmission from index and secondary cases. We found that current reporting delays are adequate for hepatitis A and B control. However, reporting delays should be reduced by a few days to improve measles and mumps control, by at least 10 days to improve shigellosis control, and by at least 5 weeks to substantially improve pertussis control. Our method provides quantitative insight into the required reporting delay reductions needed to achieve outbreak control and other transmission prevention goals.

## Abstract 2

In the Netherlands, reporting is timely for hepatitis A and B prevention but needs to be faster for measles, mumps, pertussis, and shigellosis prevention.

Timely reporting of infectious disease cases enables public health authorities (PHAs) to take effective action to prevent outbreaks by reducing disease transmission in a population. Therefore, many countries have notification systems for reporting infectious diseases to local PHAs. However, delays in the chain of reporting are inevitable. Figure 1 shows a schematic notification chain with its various delay links. The causes and durations of these links have diverse origins that must be individually analyzed to find possible ways of reducing them but only if reducing the total reporting delay (*D_OR_* in Figure 1) proves worthwhile. Although any reduction of reporting delay provides individual benefit, aiming for overall reduction of the reporting delay makes sense at population level only if a given goal for improving outbreak control can be achieved. Therefore, the question arises as to whether PHAs should spend time, money, and effort to achieve effective improvement of the total reporting delay. 

**Figure 1:**
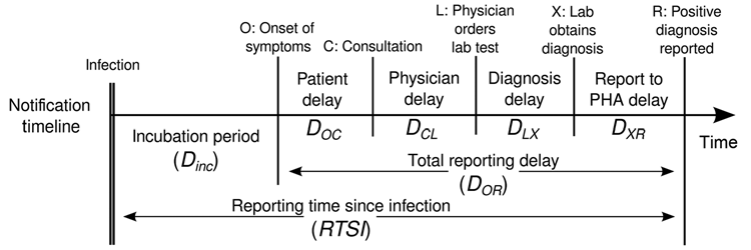
Timeline for chain of disease reporting, the Netherlands. Lab, laboratory; PHA, public health authority.

Previous studies have found that for most diseases, the reporting delays are too long to prevent directly infected contacts from spreading the disease (*1*–*3*). Few studies have taken into account the full-time distribution of events in the reporting chain (*4*,*5*), and there has been no quantitative assessment of the effect of reporting delays on outbreak control. Moreover, assessing reporting timeliness by considering only time delay does not enable a comparison among different diseases because they generally develop over different timescales.

In this article, we show how to quantify reporting timeliness for outbreak control by calculating the proportion of infections expected to be caused by index cases and by their corresponding secondary cases (*6*) until the moment the index case is reported to a PHA. This approach enables not only quantitative assessment of the effect of reporting delay reduction for a particular disease but also comparison of reporting timeliness among different diseases. Our models take into account reporting delay distributions, generation (serial) interval distributions, and distributions of symptom-onset period. We used notification data for 6 infectious diseases reported to the Netherlands notification system to evaluate the current reporting timeliness and reporting delay reductions needed to substantially affect outbreak control. The effect of a reporting delay on new infections acquired from an index case (and subsequent secondary cases) indicates to public health officials the potential value of attempting to reduce the total reporting delay and the extent to which it may need to be done.

## Methods

For evaluation of reporting timeliness, we selected 6 notifiable diseases that are transmitted person to person and for which sufficient data on total reporting delays (*D_OR_*) are available in the notification system used in the Netherlands (OSIRIS): hepatitis A, hepatitis B, measles, mumps, pertussis, and shigellosis. For 5 of those diseases, we obtained data for day of symptom onset and day of reporting to the PHA for all cases reported from July 2003 through December 2011. The other disease, mumps, was notifiable in the Netherlands until 1999, when it was dropped from the notifiable list because of a decreased number of cases. However, after a resurgence in the number of cases in 2008, mumps was reintroduced as a notifiable disease in OSIRIS in 2009. Therefore, no mumps data were available during 1999–2008. We included mumps in our analysis because of its high incidence even though control measures are limited, similar to the situation with pertussis. To model the reporting delay for each disease, we fitted analytical log-normal distributions to the OSIRIS data. We also used log-normal distributions that fit serial interval and time-to-symptom onset ranges found in the literature (Table 1).

**Table 1:** Parameters for reporting delay models, by disease*

Disease	Serial interval distribution, median days (SD)	Symptom onset distribution, median days (SD)	Reporting delay distribution, median days (SD)	Reproduction number, R (range)†	References
Hepatitis A	27.5 (4)	28 (9)	8.6 (11.9)	3.33 (3–4)	(*7*–*10*)
Hepatitis B	47.5 (20)	80 (35)	14.7 (24.3)	1.75 (1–2.5)	(*7*–*9*,*11*)
Measles	11.6 (2.4)	11.5 (2.5)	9.0 (12)	8 (8–30)	(*7*–*10*,*12*)
Mumps	19.1 (5.4)	19.5 (2.3)	9.0 (13.8)	5.5 (4–7)	(*7*–*10*)
Pertussis	16 (13)	9 (2.5)	40.8 (24.4)	5.5 (5–6.5)	(*7*–*10*,*13*,*14*)
Shigellosis‡	5 (3.5)	2.5 (1.5)	14.6 (13.8)	3.5 (2–5)	(*7*–*9*)

*All distributions are fitted to log-normal distributions with medians and standard deviations as indicated. Reporting delay distribution of pertussis is an exception, which is fitted to a gamma distribution. †The reproduction numbers are those used for outbreak control calculations, and the ranges in brackets are those found in the literature. ‡For shigellosis, an average transmission period (serial interval distribution) of 1 wk (median 5 d) was assumed, although in practice shedding continues after that.

The course of infection for each disease has its own characteristic time scale (latent, infectious, and symptomatic periods). Thus, a 1-week delay might have a substantial effect on control of a slowly progressing disease such as hepatitis A but not on a rapidly progressing disease such as shigellosis. Moreover, reporting itself also has its own time scale because of various factors behind each link in the reporting chain. Therefore, for timeliness of case reporting to be assessed and compared for various diseases, timeliness needs to be evaluated in terms of the number of infections that could not be prevented because of the delay, rather than in terms of the actual time taken to report cases.

When a case is reported, regional PHAs implement mostly case-based interventions. These interventions are intended to prevent transmission from the reported case and from secondary cases that may have been acquired from the index case. Secondary cases are identified by contact tracing. For this reason, for each disease we first calculated the proportion of expected infections produced by an index case (PIR1) until the moment the index case in question is reported to the local PHA. We then calculated the proportion of expected infections produced by each secondary case produced by a reported index case (PIR2) until the moment the index case in question is reported to the local PHA. Throughout this study we refer to an index case as any case that is reported because of a positive diagnosis and a case that has not yet been traced as a secondary case when reported (i.e., all primary cases that may result in clusters). For every calculation, we considered the hypothetical intervention in which contact tracing and stopping of transmission occur instantly when the index case is reported. Such a rapid response is not realistic, but the estimate provides an upper limit for outbreak control potential as determined by reporting speed. The calculations were performed by using scripts written in Python programming language (https://www.python.org). Below is an introductory explanation of our calculations; further details and explicit formulas are provided in the Technical Appendix).

### Calculation of PIR1, a 1-Generation–based Response

**Figure 2:**
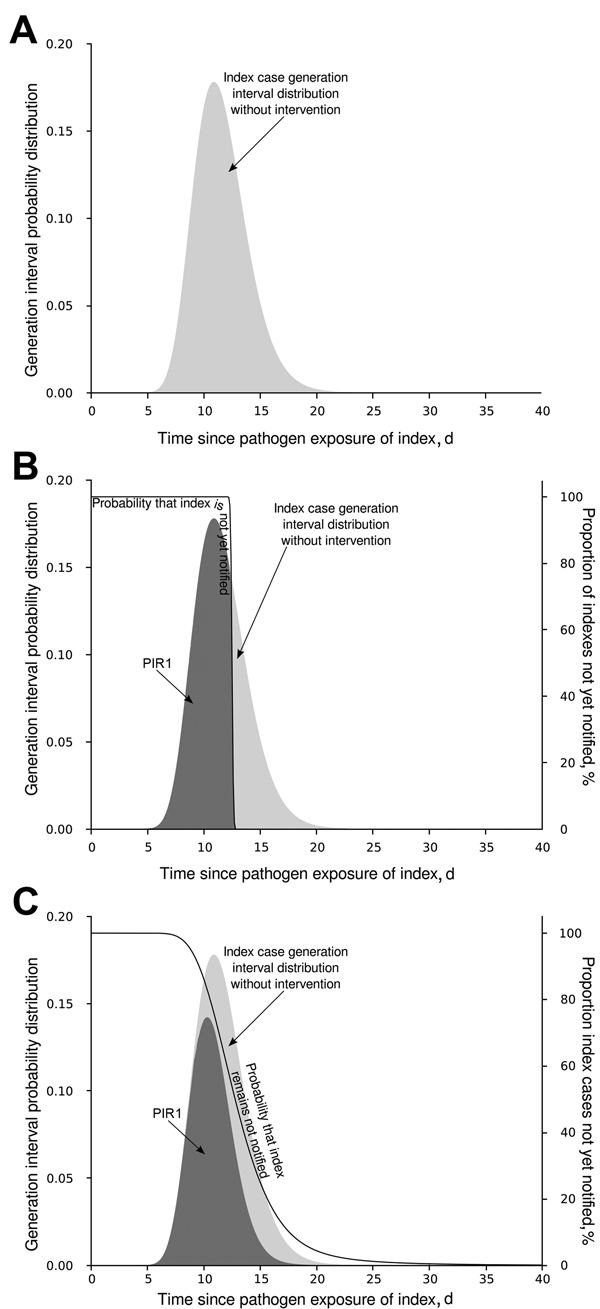
Schematic modification of PIR1. A) Generation interval distribution of an index case as function of time since the index case acquired the pathogen. Without notification and intervention, the proportion of infections expected by the index case is 1, the light gray area under the curve. B) How the generation interval distribution is modified, assuming that all index cases are notified and stopped exactly 13 days after exposure to the pathogen. C) How the average generation interval is modified when index cases are notified and stopped according to a time distribution. Dark gray shading indicates the PIR1 value for each situation. The black line indicates the proportion of index cases not yet notified (right y-axis), equivalent to the probability of an index case not yet being notified in each situation. PIR1, expected proportion of cases caused by index case at notification.

The generation interval is the time elapsed from the moment an infector acquires a pathogen until he/she infects another host. The distribution of the generation interval time then indicates the average infective profile since an index case acquired the pathogen (*15*). Figure 2, panel A, shows distribution of an index case generation interval: initially a period when no infections take place (latent period); then as time passes, the profile rises (beginning of infectious period) to a peak (most infections occur at this moment) and later declines, leading to the end of the infectious period. We considered the reporting time elapsed since infection (RTSI) to be the addition of the symptom onset time (*D_INC_*) and the reporting delay (*D_OR_*) (Figure 1). Figure 2, panel B, shows PIR1 in the extreme case that RTSI is fixed at 13 days for every infector, meaning that the probability of not being reported before RTSI is 1 and after RTSI is 0. The proportion of expected infections produced by an index case (PIR1) until reporting time is then provided by the area under the generation interval curve weighted by the probability of not yet being reported, equivalent to the percentage of nonreported cases. However, times for symptom onset and reporting delays vary from person to person, and RTSI then becomes a distribution. This distribution smooths out the step-like probability of any infector not being reported at a given time (Figure 2, panel C), which consequently smooths out how the generation interval must be weighted for an appropriate PIR1 calculation.

### Calculation of PIR2, a 2-Generation–based Response

**Figure 3:**
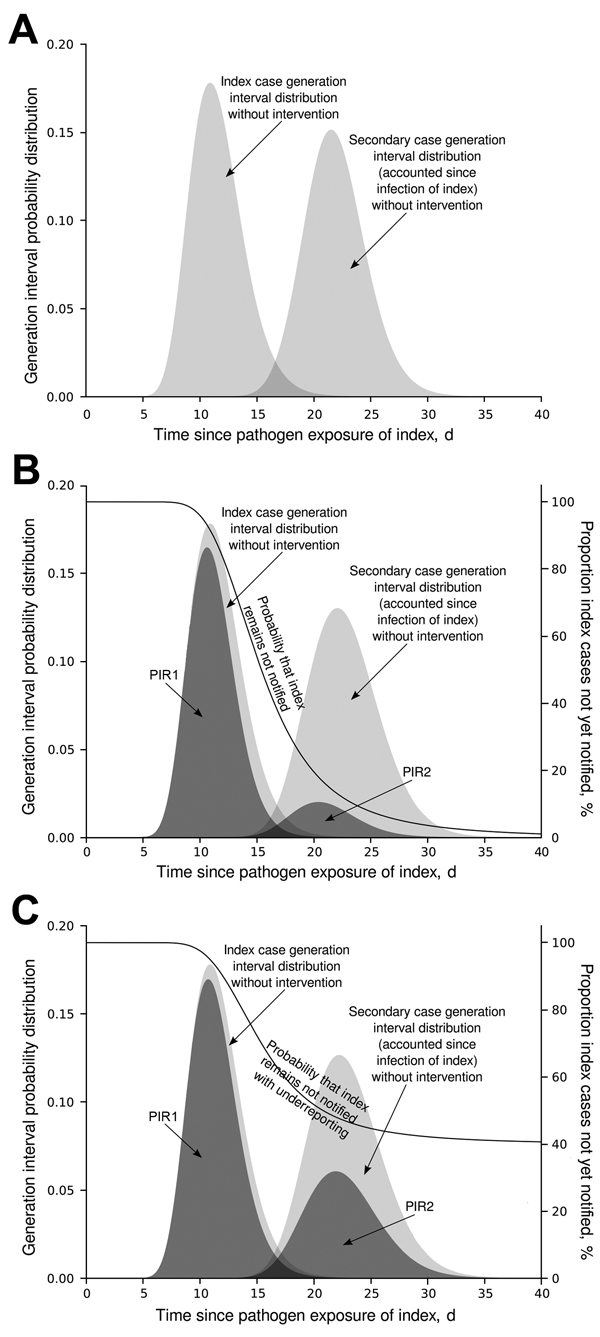
Schematic modification of PIR2. A) Generation interval time distributions of index and secondary cases, from the moment of exposure of the notified index case. PIR2 is represented by the area under the second generation interval distribution, which is 1 in the absence of notification/intervention. B) PIR1 and PIR2 values when index cases are notified and stopped together with their secondary cases, according to a time distribution. C) How PIR values in panel B are modified by 40% underreporting. Dark gray shading indicates PIR1 and PRI2 values. The black line indicates the proportion of index cases not yet notified (right y-axis), equivalent to the probability of an index case not yet being notified in each situation. PIR1, expected proportion of cases caused by index case at notification; PIR2, expected proportion of new infections caused by secondary cases before index case is notified.

The calculation of the proportion of expected infections produced by secondary cases at the time when their corresponding index case is reported (PIR2) is conducted in the same way as that of PIR1, but a 2-generation interval is used instead of a standard generation interval. A 2-generation interval is constructed by subsequently adding 2 standard generation intervals. The 2-generation interval distribution indicates the average second-generation infective profile as time passes since the index case acquired the pathogen (Figure 3, panel A). PIR2 is then provided by the area under the 2-generation interval curve weighted by the probability of the index case not yet being reported (Figure 3, panel B).

At an early stage, an outbreak is controlled (i.e., prevalence begins to decline) by implementing a case-based intervention that can stop transmission early enough so that the number of cases produced per infector is <1 (*6*,*16*,*17*). The number of cases produced per index case is calculated by multiplying PIR1 times the reproduction number (R) of the disease in question. The number of cases produced per secondary case is PIR2 multiplied by the reproduction number. Hence, for each disease, we assumed that the conditions for outbreak control are PIR1<1/R and PIR2<1/R. In addition, given that PIR2 involves 2 generations, we considered the alternative (more restrictive) outbreak control condition PIR2<1/R^2^. To evaluate the status of the current reporting timeliness of the 6 diseases, we compared our PIR1 and PIR2 results with the outbreak control conditions.

To assess the potential for improvement by reducing current reporting delays for each disease, we studied how much various reporting delays influence PIR1 and PIR2 and calculated the reporting delay reductions needed to reach outbreak control conditions. PIR1 and PIR2 were highly dependent on the reporting delay median but not so regarding standard deviations within the range matching actual reporting delay distributions (Technical Appendix Figure). Therefore, for simplicity, we present our reporting delay analysis results as medians of 1–60 days under the assumption that standard deviations are equal to the medians**.** For each disease, we evaluated the ratio at which PIR1 and PIR2 are reduced after the reporting delay median is reduced by 1 day, as extracted from the OSIRIS database. These reduction ratios enabled us to determine those diseases for which reduction of reporting delays would most prevent further transmission.

### Underreporting

Our calculations of PIR1 and PIR2 were made with the assumption of 100% reporting compliance. However, a proportion of cases are not reported (and might include asymptomatic cases). From an outbreak control point of view, underreporting can be tackled by assuming that there are only reported cases, each producing an increased average number of infections to account for the contribution to disease transmission from the cases that are not reported (Figure 3, panel C). PIR1 and PIR2 are modified as follows: PIR(underreported) = PIR × (1 − proportion.underreported) + proportion.underreported. Therefore, there is a maximum limit for underreporting beyond which it is not possible to satisfy the outbreak control condition R × PIR(underreported) <1. We calculated this limit for the studied diseases by assuming instantaneous reporting at the day of symptom onset. Although this assumption is not realistic, it provides an upper limit estimate for underreporting if outbreak control is desired.

### Vaccination Coverage

Many diseases are preventable by vaccination; among the 13 diseases in the National Immunization Program vaccination schedule for the Netherlands are hepatitis B, measles, mumps, and pertussis. Consequently, part of the population might be protected by vaccine-induced immunity. When considering that individually targeted interventions are implemented effectively, the minimum vaccination coverage needed to achieve herd immunity is reduced. We calculated this reduction for the 6 studied diseases by first considering the current reporting delays and the outbreak control condition [R(1 − coverage)]^2^ × PIR2<1. From this condition we derived the reduced minimum vaccination coverage needed for outbreak control and compared it with the standard vaccination coverage needed for achieving herd immunity (coverage>1 – 1/R).

### Model Parameters

We fitted log-normal distributions to frequency distribution of reporting delays as extracted from OSIRIS. We performed fitting with a Kolmogorov-Smirnov minimization and a 0.05 significance level by using the program Mathematica version 8.0 (http://www.wolfram.com/mathematica/). Table 1 shows the parameters we found and used in our models for each disease.

Because generation intervals are difficult to observe, we used the serial interval as a proxy for the generation interval (*18*) and assumed equivalence. The serial interval is the time between symptom onset of an index case and symptom onset of a secondary case. For each disease we extracted information on incubation period distribution, serial interval, and reproduction number from the published literature (Table 1). Most data on incubation periods and serial intervals in the literature are provided as a range with a relevant value (average, median, or mode). Therefore, we constructed the distributions by finding parameters for which a log-normal distribution would present the relevant value found in the literature, and the 2.5th and 97.5th percentiles would correspond to the ranges found in the literature. Log-normal distributions are easy to handle, and there is evidence favoring them as incubation period distributions (*19*).

## Results

### Current Reporting Timeliness

**Figure 4:**
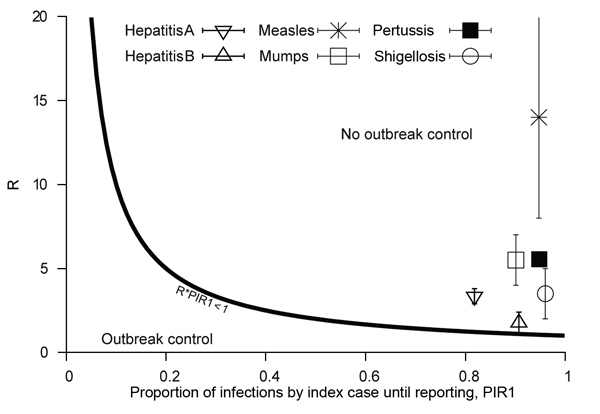
Timeliness of current reporting speed in the Netherlands, evaluated considering interventions applied to reported index cases only. The outbreak control condition is R × PIR1<1. Diseases that lie in the outbreak control areas are those for which reporting speed is timely enough to enable outbreak control. PIR1, expected proportion of cases caused by index case at notification; R, reproduction number.

Current reporting timeliness in the Netherlands is shown in Figure 4; calculations are based on data from the Netherlands and consider interventions applied only to the reported index case. For most diseases, the expected proportion of infections produced until reporting is >90%. The expected proportion of infections is lower for hepatitis only; however, this proportion is still high at >80%. Therefore, if an index case is instantly removed as a source at the moment of reporting, in general <10% secondary infections are prevented, which renders such an intervention rather ineffective. Even fewer infections can be prevented if underreporting is considered. All diseases shown in Figure 4 lie above outbreak control condition.

**Figure 5:**
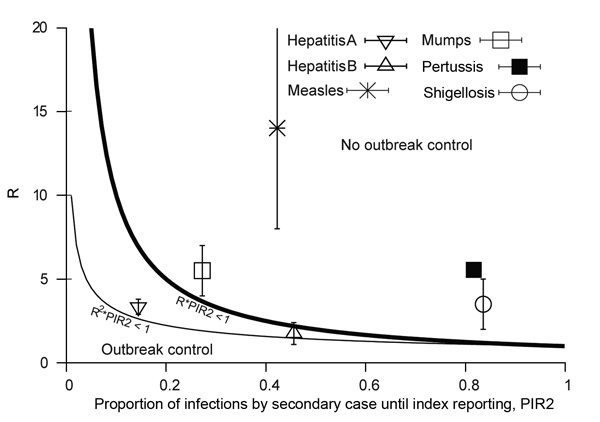
Timeliness of current reporting speed in the Netherlands, evaluated considering interventions applied for reported index cases and their secondary cases. The lower outbreak control condition is R^2^ × PIR2<1, assuming index cases are reported too late to stop any secondary infection (i.e., PIR1 = 1 always). The upper outbreak control condition R × PIR2<1, which is the most relaxed condition, assumes an extreme situation that index cases have not caused more infections than secondary cases (PIR1 = PIR2). In practice, the outbreak control condition lies in between these 2 conditions. Diseases that lie in the outbreak control areas are those for which reporting speed is timely enough to enable outbreak control. PIR1, expected proportion of cases caused by index case at notification; PIR2, expected proportion of new infections caused by secondary cases before index case is notified; R, reproduction number.

When interventions are also applied to secondary cases produced by a reported index case, the interventions become more effective. The expected proportion of infections produced by secondary cases, PIR2, for the 6 diseases is shown in Figure 5. Hepatitis A lies close to the lower outbreak control condition, indicating that its current reporting speed should be timely enough to keep it under control. The same indication applies to hepatitis B, which also lies below the upper outbreak control limit, despite its intermediate PIR2 values. PIR2 for measles is intermediate, which places the disease far outside the area in which control is possible. PIR2 is low to intermediate for mumps, but the reproduction number for mumps is smaller than that for measles, which places the disease close to the upper outbreak control condition. Pertussis and shigellosis remain in the region of high PIR2 values, meaning that secondary cases may have already produced most infections at the moment that the index case is reported, thereby limiting the effectiveness of outbreak control by means of contact tracing.

### Room for Improving Reporting Timeliness

**Figure 6:**
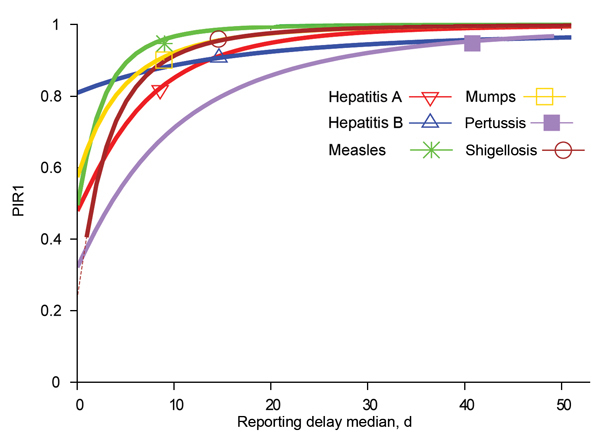
PIR1, depending on reporting delay median for the indicated diseases and assuming standard deviation equal to median value. Thick lines show reporting delay medians for which there is no outbreak control. Thin dashed lines would show reporting delay medians that bring diseases within the outbreak condition (R × PIR1<1), but they are not present because even with extremely short delays it is not possible to fulfill the condition with the studied diseases (except shigellosis). Symbols indicate PIR1 evaluated with current reporting delay data. PIR1, expected proportion of cases caused by index case at notification; R, reproduction number.

Interventions applied only to index cases are rather inefficient, even with the swiftest reporting. Figure 6 shows that even when a case is (unrealistically) reported on the same day of symptom onset (median = 0), PIR2 values are above their respective outbreak control limit for 5 of the 6 studied diseases. This finding is because of the proportion of expected secondary infections an index case produces while asymptomatic (*6*). The exception is shigellosis, but for this disease already a median reporting delay of 1 day would be too late for implementing outbreak control. In general, only short reporting delays of ≈3 days would enable substantial reduction of PIR1 for the 6 diseases. Moreover, with current reporting delays, the largest PIR1 reduction ratio achieved by reducing current delays by 1 day is 2.6% (for hepatitis A) (Table 2).

**Table 2:** Effects of reducing reporting delays, by disease*

Effect	Hepatitis A	Hepatitis B	Measles	Mumps	Pertussis	Shigellosis
Current PIR1 (PIR1 at symptom onset)	0.818 (0.480)	0.907 (0.810)	0.947 (0.495)	0.901 (0.574)	0.948 (0.322)	0.960 (0.230)
Current PIR2 (PIR2 at symptom onset)	0.145 (0.019)	0.456 (0.320)	0.423 (0.006)	0.273 (0.005)	0.817 (0.067)	0.836 (0.056)
PIR2 reduction ratio by reducing delay in 1 d	9.7%	1.5%	12.6%	13.5%	0.7%	2.4%
Reporting delay median needed for PIR2 = 1/R	17 d	42 d	5 d	8 d	4.5 d	3 d
Reporting delay median needed for PIR2 = 1/R^2^	8 d	1 d	2 d	3 d	Not possible	1 d
Underreporting beyond which outbreak control is not possible	29%	1.5%	12%	18%	12%	25%
Reduction of vaccination coverage for herd immunity	70%	64%	8%	20%	2%	4%

*PIR values at symptom onset show a theoretical minimum, achievable by stopping transmission instantly at symptom onset. PIR1, proportion of expected infections produced by an index case; PIR2, proportion of expected infections produced by each secondary case produced by a reported index case.

**Figure 7:**
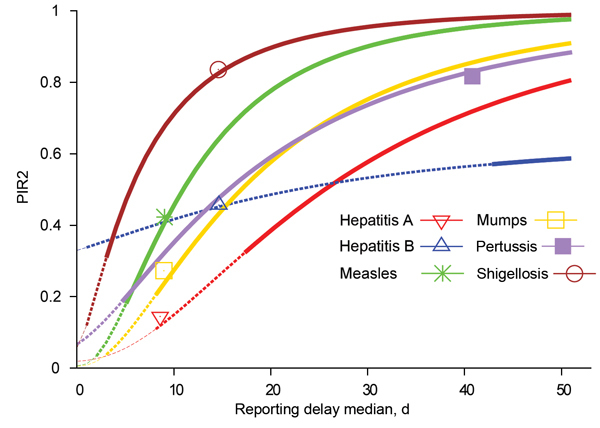
Expected proportion of infections caused by secondary cases before reporting of their index case, depending on reporting delay median for the indicated diseases and assuming standard deviation equal to median value. Thick lines show reporting delay medians for which there is no outbreak control. Intermediate-width dashed lines show reporting delay medians that bring diseases within the upper outbreak condition (R × PIR2<1). Thin dashed lines show reporting delay medians bringing diseases under the lower outbreak control condition (R^2^ × PIR2<1). Symbols indicate PIR2 evaluated with current reporting delay data. HepA, hepatitis A; hepB, hepatitis B; PIR2, expected proportion of new infections caused by secondary cases before index case is notified; R, reproduction number.

However, for some diseases, the efficiency of applying interventions also to traced contacts can be substantially increased by reducing reporting delays. Relevant results are summarized in Table 2. Figure 7 shows that for sufficiently short reporting delay medians, the upper outbreak control condition could eventually be satisfied for all 6 diseases. If the median reporting delay for hepatitis A were 8 days, the lower, more restrictive, outbreak control condition could be satisfied. Outbreak control for measles and mumps would need reporting delay medians of 2 and 3 days, respectively. For hepatitis B and shigellosis, reporting would need to be almost instantaneous (1-day delay); for pertussis, the lower outbreak control condition could not be satisfied even if reported the same day as symptom onset. The PIR2 reduction ratio is high for hepatitis A, measles, and mumps, indicating that substantial improvement can be achieved with reporting delay reductions of 1 (or a few) days. However, little improvement is expected with a small reduction of reporting delays for hepatitis B, pertussis, and shigellosis. Table 2 shows that with underreporting >30%, outbreak control for all 6 diseases is not possible. Table 2 also shows that only for hepatitis A and hepatitis B would the immunization coverage needed to achieve outbreak control be substantially reduced, because of individually targeted interventions with the current reporting speed.

## Discussion

Public health agencies are responsible for organizing a swift course of action from disease onset to notification and intervention. The general assumption is that the shorter the delay, the better the control response. We quantitatively assessed the potential of individual-based interventions by PHAs for minimizing or preventing outbreaks by calculating the expected proportion of infections caused by index (PIR1) and secondary (PIR2) cases until reporting of the index case. For hepatitis A, measles, mumps, pertussis, and shigellosis, PIR1 was >90% (Figure 3), but for hepatitis B, PIR1 was 82%. This finding indicates that interventions aimed only at notified index cases are always too late to substantially prevent future disease transmission. Therefore, effective control requires contact tracing and stopping transmission from contacts. Even if reporting delays were reduced to a couple of days, interventions targeting index cases only are not enough to achieve outbreak control conditions.

The expected proportion of infections caused by secondary cases, PIR2, differs substantially among different diseases. Current reporting delays for hepatitis A and hepatitis B lead to PIR2 values that are within outbreak control limits (Figure 5), probably because of the long incubation period for each disease. Although incubation periods for pertussis and shigellosis differ greatly, reporting for these diseases is far from meeting outbreak control conditions (Figure 5), probably resulting partly from patient and physician reporting delays because of nonspecific symptoms. Measles and mumps appear in the middle of Figure 5, despite their relatively short incubation periods, probably because they produce specific symptoms. For measles and mumps, a combined effort to lower their reproduction number and shorten notification delay might bring their reporting within outbreak control conditions. Lower reproduction numbers move diseases to a lower position in Figures 4 and 5. This lowering can be achieved by interventions at population or group levels (e.g., vaccination or hygiene/behavior changes). Areas with high baseline vaccination ratios against measles or mumps (boosters included) are closer to meeting outbreak control conditions than those with low baseline vaccination ratios and need less drastic delay reductions to achieve the same effect when interventions are applied to secondary cases.

Whether a PHA response to a reported case is considered timely depends on the goal set for the intervention and the balance between the benefit and efforts spent on reducing reporting delays. PIR2 decreases substantially with a reporting delay reduction of a few days for hepatitis A, measles, and mumps. This finding suggests that efforts to reduce reporting delays for these diseases would be worthwhile because doing so would increase the effectiveness of interventions applied to secondary cases. However, using resources to reduce reporting delays for hepatitis B, pertussis, and shigellosis would not be worthwhile for outbreak control purposes because a substantial increase of prevented infections could be achieved only with extreme reporting delay reductions (pertussis and shigellosis) or none at all (hepatitis B).

In the context of transmission of infection, calculations of PIR1 and PIR2 provide an objective measure for the timeliness of reporting and interventions. We focused on outbreak control as a goal (reducing reproduction number to <1), but the method we described can also be used to assess reporting delays with another goal in mind. For example, for an extremely serious disease, the goal might be to reduce PIR1 and PIR2 to the smallest acceptable limit.

Maximum limits of underreporting that would allow for any possibility of outbreak control are rather small (Table 2). Therefore, in addition to being timely, reporting must also be very complete.

The median is a robust characteristic of a dataset because it is not influenced largely by outliers. Therefore, medians have been used in many studies to compare data on factors such as latent periods, serial intervals, and notification delays but without taking into account the shape of the distributions (*1*–*3*). It is reassuring that we found that calculations of PIR1 and PIR2 depend mainly on medians and not on the standard deviations of reporting delay distributions.

We have quantitatively assessed the outbreak control potential of PHA responses on the basis of the timeliness of the current reporting delays of hepatitis A, hepatitis B, measles, mumps, pertussis, and shigellosis in the Netherlands. We used the expected proportion of infections caused by index and secondary cases until reported to the local PHA (PIR1 and PIR2). These disease-specific quantities provide a powerful tool for setting goals for reporting speeds, not only for outbreak control but also for evaluation of individual-based interventions with other aims, such as partially reducing infections or completely stopping transmission.

Technical Appendix Details and explicit formulas for calculations of effects of reporting delays on outbreak control.
