# Supplementary material for: Quantifying Reporting Timeliness to Improve Outbreak Control
Source: Emerg Infect Dis. 2015 Feb;21(2):209–16. doi: 10.3201/eid2102.130504 (PMC4313625; doi:10.3201/eid2102.130504)
Supplement: Technical Appendix — Details and explicit formulas for calculations of effects of reporting delays on outbreak control. [file 13-0504-Techapp-s1.pdf]

Article DOI: http://dx.doi.org/10.3201/eid2102.1430504

Quantifying Reporting Timeliness to Improve Outbreak Control

Technical Appendix

Mathematical Framework

Fraser et al. (*1*) introduced a framework to evaluate how controllable any infectious disease outbreak by calculating the proportion of new infections caused by an index until symptom onset, and subsequently evaluating outbreak control conditions by assuming that public health control measures take place immediately. Inspired by this idea, we propose a framework to evaluate timeliness by calculating the proportion of new infections caused by an infector until the moment the index case is reported to a PHA. By assuming that control measures take place at that moment, the proportion of potentially prevented cases can be assessed and outbreak control conditions can be evaluated. To set the layout of our framework we define the following:

- R, reproduction number: expected number of new infected cases generated directly by one infector during his entire infectious period.
- τ, infection-age of an individual: time elapsed since the moment he/she is infected.
- g, Generation interval distribution: probability distribution of time interval between infection of an index case and infection of a secondary case.
- $\theta(\tau)$, expected proportion of infections produced until infection-age τ. Particularly and most important in the present study, we denote:
    - θ_{r1} (PIR1 in the main manuscript), the expected proportion (or number if multiplied

by R) of infections produced by an index case until he/she is reported to the regional PHA.

    - θ_{r2} (PIR2 in the main manuscript), the expected proportion (or number if multiplied by R) of infections produced by secondary cases until the index case who produced them is reported to the regional PHA.

- $f_{inc}(\tau)$, incubation period distribution: probability distribution of time between infection and symptom onset.
- $N_1(\tau)$, reporting delay distribution: distribution of time between onset of symptoms and reporting to PHA.

One-generation based response

We assumed transmission from the reported case is instantaneously stopped from the moment of reporting. We calculated θ_{r1} (PIR1 in the main document) by integrating the effective first generation interval $g_{1e}(\tau)=g(\tau)(1-[N_1*f_{inc}](\tau))$ in τ (Fig. 2C in the main document). As defined above, $g(\tau)$ is the conventional generation interval distribution, $f_{inc}(\tau)$ the incubation period distribution and $N_1(\tau)$ the reporting delay distribution. The convolution

$$[N_1*f_{inc}](\tau) = \int N_1(t) f_{inc}(\tau-t)dt$$

represents the probability of an index being reported at infection-age τ.

Two-generation based response (contact tracing)

We considered the hypothetical intervention where contacts (secondary cases) are traced and stopped (together with their source person) instantly from transmitting onwards at notification time of the index case. We calculated θ_{r2} (PIR2 in the main manuscript) by integration in τ of the effective second generation interval $g_{2e}(\tau)=g_2(\tau)(1-[N_1*f_{inc}](\tau))$ (Fig. 3B.in

the main document), with $[N_1*f_{inc}](\tau)$ as defined above. Here $g_2(\tau)$ is the generation interval distribution of secondary cases as function of infection-age of their index case and is computed by the convolution $[g*g_{1e}](\tau)=\int g(t)g_{1e}(\tau-t)dt$, where $g_{1e}(\tau)$ is the effective first generation interval defined above.

Influence of reporting delay spread (standard deviation) on expected proportion of infections

We calculated θ_{r1} and θ_{r2} values (PIR1 and PIR2 in the main document, respectively) using various reporting delay distributions. This allowed us to study how much reporting delay variations influence the values of θ_{r1} and θ_{r2}.

To cover a range of various reporting delay distributions we parametrised in terms of median and standard deviation (SD) and used a set of these parameters. Notification median delays ranged from 1 to 60 days, in steps of 1 day. Standard deviations were chosen as multiples 0, 0.5, 1, 1.5 and 2 of the median. The Appendix Figure shows that the expected proportion of infections caused by index (θ_{r1}, or PIR1) and by secondary cases (θ_{r2}, or PIR2) are highly dependent on reporting delay medians. However, the figure also shows that θ_{r1} and θ_{r2} do not sensitively depend on standard deviation values within the range matching actual reporting delay distributions (SD=0.5-1.5*median, see Table 1 in the main document).

Reference

1. Fraser C, Riley S, Anderson RM, Ferguson NM. Factors that make an infectious disease outbreak controllable. Proc Natl Acad Sci U S A. 2004;101:6146–51. PubMed http://dx.doi.org/10.1073/pnas.0307506101

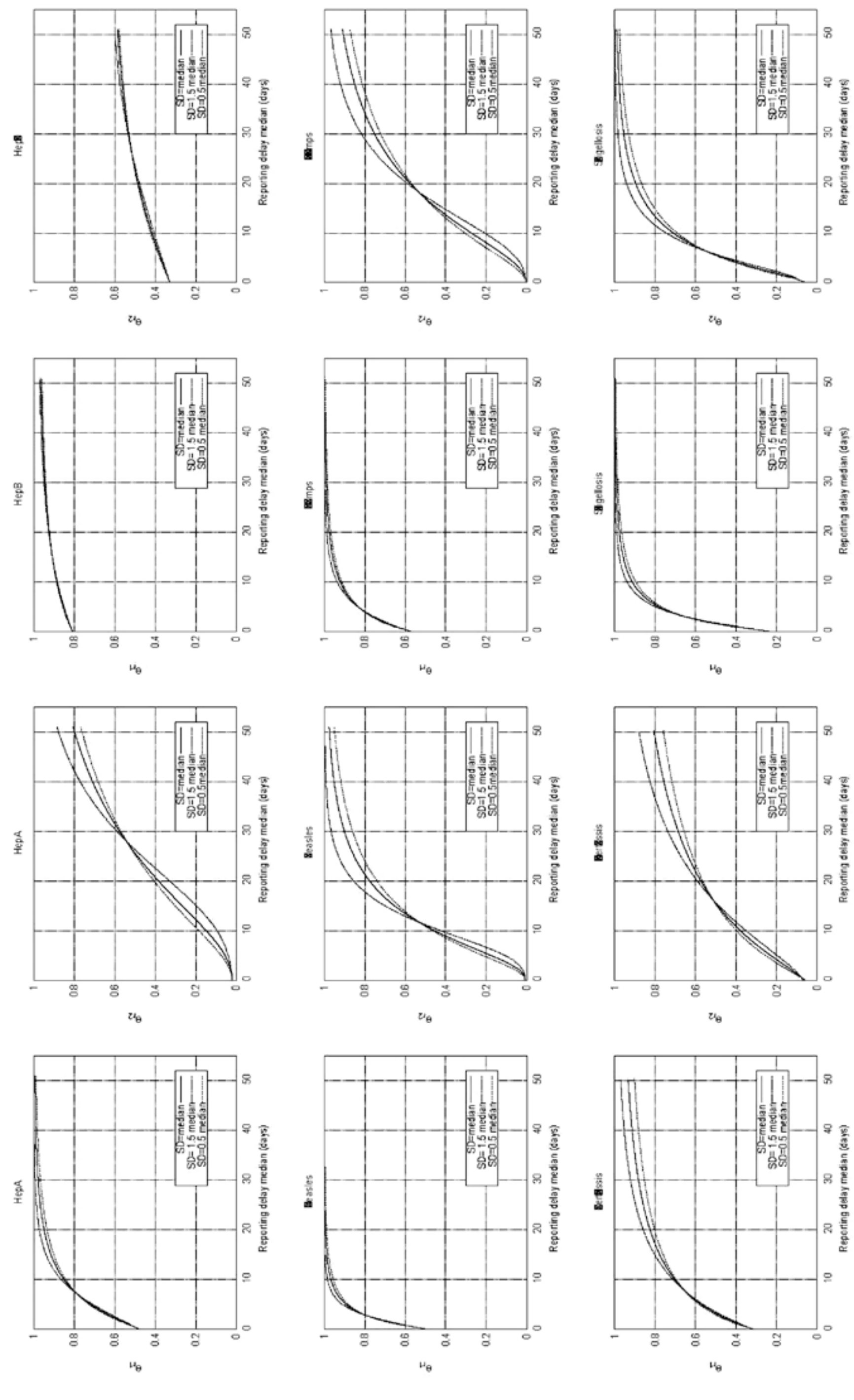

Appendix Figure. Expected proportion of infections caused by an index case (θ_{r1}, or PIR1 in the main manuscript) and by secondary cases (θ_{r2}, or PIR2 in the main manuscript) until reporting of the index case at PHA, for each studied disease as indicated. θ_{r1} and θ_{r2} are shown as a function of reporting delay median in days. The various line types indicate results for using various standard deviation values for the reporting delay distribution, as indicated in the legend.
